# Optimization of Intercropping Modes in *Placodon grandiflorus*

**DOI:** 10.1002/pei3.70036

**Published:** 2025-02-16

**Authors:** Pengfei Liu, Jiannan Ma, Juan Yu, Meixi Zhang, E. Qiao, Yang Cao, Ying Zhang, Xiaoqin Wang, Xin Jia

**Affiliations:** ^1^ School of Pharmacy Inner Mongolia Medical University Hohhot China

**Keywords:** continuous cropping, intercropping, physiological and biochemical indicators, *Platycodon grandiflorus* (Jacq.) a. DC, saponin content

## Abstract

Intercropping enhances plant growth, increases yield, and boosts the accumulation of secondary metabolites. *Platycodon grandiflorus* ( *P. grandiflorus* ), a traditional Chinese medicinal herb, has limited research available regarding its intercropping practices. We aimed to (1) examine the changes in the physiological and biochemical indicators of plant growth during the intercropping process of *P. grandiflorus* , (2) assess the quality of *P. grandiflorus* when intercropped with different crops, and (3) evaluate the optimal intercropping mode for *P. grandiflorus.* This study utilized the two‐year seedlings of *P. grandiflorus* as the test material in a field study. The intercropping treatments included *P. grandiflorus* monoculture (JG‐JG), intercropping with *Achyranthes bidentata* (JG‐NX), *Saposhnikovia divaricata* (JG‐FF), *Adenophora stricta* (JG‐SS), *Zea mays* (JG‐YM), *Setaria italica* (JG‐GZ), and *Glycine max* (JG‐DD). We investigated the effects of these different intercropping modes on the growth, physiological and biochemical indicators, and the accumulation of five saponins in *P. grandiflorus* at various growth and development stages. Compared with JG‐JG, the chlorophyll and the MDA contents significantly increased and decreased, respectively, in the JG‐YM, JG‐DD, and JG‐NX treatments. All the three treatments enhanced the biomass and exhibited the higher levels of antioxidant enzyme activity and osmoregulatory substance content. JG‐YM and JG‐SS significantly (*p* < 0.05) improved the quality of *P. grandiflorus* , with JG‐SS intercropping notably maintaining a high content of platycodin D. The results of this study provide a scientific basis for optimizing intercropping planting systems and advancing the sustainable development of the traditional Chinese medicine industry.

**Abbreviations**
CAT: catalase
JG‐DD: intercropping *P. grandiflorus* and *Glycine max*

JG‐FF: intercropping *P. grandiflorus* and *Saposhnikovia divaricata*
JG‐GZ: intercropping *P. grandiflorus* and *Setaria italica*

JG‐JG:
*P. grandiflorus* monoculture
JG‐NX: intercropping *P. grandiflorus* and *Achyranthes bidentata*

JG‐SS: intercropping *P. grandiflorus* and *Adenophora stricta*
JG‐YM: intercropping *P. grandiflorus* and *Zea mays*

MDA: malondialdehyde
POD: peroxidase
SOD: superoxide dismutase

## 1 Introduction

Continuous cropping involves planting the same crop in the same field for consecutive years. Despite normal management practices, this method can lead to the reduction in yield, the deterioration in quality, and the decline in soil fertility after repeated planting of the same or closely related crops (Ma et al. 2023). Continuous cropping obstacles have become a widespread issue globally, affecting various crops, from the grain crops such as wheat, corn, rice, and soybeans to the economic crops including vegetables, fruits, and flowers in facility agriculture, each experiencing varying degrees of these obstacles. However, over 40% of Chinese medicinal materials require manual cultivation, and approximately 70% of rhizome‐based Chinese medicinal materials encounter significant obstacles to continuous cropping during cultivation (Miao et al. 2024). The challenges primarily include the decrease in germination rate, the increase in mortality rate, and the rise in disease incidence during the growth period. Consequently, there is a decline in the quality of traditional Chinese medicine, which can adversely affect the effectiveness of medicinal ingredients and may even increase toxicity, thereby influencing the overall efficacy.

Several methods can mitigate the obstacles to continuous cropping, including crop rotation and intercropping. As a cornerstone of traditional Chinese agriculture, intercropping has offered certain benefits such as increased yield, enhanced nutrient resource utilization, and improved pest and disease control through biodiversity, which also bolsters the ecological stability of farmlands (Zeeshan Ul Haq et al. 2023; Zhang et al. 2008). Studies have indicated that intercropping enhances the growth and biomass accumulation of *Hibiscus cannabinus* and *G. max* compared to monoculture and increases the antioxidant enzyme activities while reducing the malondialdehyde (MDA) content (Rehman et al. 2023). Additionally, intercropping *Pinellia ternata* with *Sedum alfredii* has been reported to promote its growth, with optimal leaf area, leaf length, leaf width, plant height, and stem thickness observed (Wang, Han, et al. 2024). The chlorophyll content of *Pinellia ternata* increases with the crown width, peaking at the crown width of 200 cm (Xue et al. 2008). Replacing and intercropping *Taizi ginseng* with local main varieties effectively reduces the replant disease and improves both the yield and quality of *Taizi ginseng* under repeated cropping conditions (Liu et al. 2022). However, research on *P. grandiflorus* remains limited, with most studies focusing on the soil analysis after continuous cropping.

Belonging to the *Campanulaceae* family and *Platycodon* A. DC, *Platycodon grandiflorus* (Jacq.) A. DC is a widely used perennial herbaceous plant in China (Feng et al. 2024). This plant is valued for its medicinal and edible properties, with *P. grandiflorus* saponins, particularly platycodin D, being the key components specified in the 2020 edition of the Chinese Pharmacopeia (Zhang et al. 2015). Recent increases in the cultivation areas for *P. grandiflorus* , driven by its diverse uses, have led to the conflicts with traditional food crop production lands (Ji et al. 2020). Consequently, the year‐round continuous planting in *P. grandiflorus* production areas has caused the inreased incidences of diseases and pests, significantly reducing the yield and quality, and compromising the authenticity of medicinal materials. This has created substantial obstacles to continuous cropping and has caused considerable losses in the production of medicinal materials (Jiang et al. 2023).

This study combined the practical research with prior studies conducted in Chengzi Township, Chifeng City, a major production area for *P. grandiflorus* in China. It focused on intercropping three local medicinal herbs, including *A. bidentata* , *S. divaricata* , *A. stricta* , and three crops, namely *Z. mays* , *S. italica* , and *G. max* . This study examined the effects of the intercropping on *P. grandiflorus* by evaluating the growth, photosynthetic characteristics, antioxidant enzyme activity, osmoregulatory substance content, and the accumulation of five saponins at various growth stages. The objective of this study was to identify the most effective intercropping methods for providing a theoretical foundation for standardized planting of *P. grandiflorus* under different intercropping modes.

## 2 Material and Methods

### 2.1 Materials and Testing Sites

The test materials consisted of the two‐year‐old seedlings of *P. grandiflorus* . The seedlings were cultivated in the experimental field situated in Chengzi Township, Songshan District, Chifeng City, Inner Mongolia Autonomous Region, China, at 118.69°N and 42.14°E, with the elevation of 872 m. The site is characterized by a temperate continental monsoon climate featuring ample sunlight, high accumulated temperature efficiency, low precipitation, and frequent droughts and winds. The average annual precipitation at this location is 355 mm.

### 2.2 Experimental Design

This study utilized a field experimental design featuring seven intercropping modes: single cropping of *P. grandiflorus* (JG‐JG), intercropping of *P. grandiflorus* and *A. bidentata* (JG‐NX), *P. grandiflorus* and *S. divaricata* (JG‐FF), *P. grandiflorus* and *A. stricta* (JG‐SS), *P. grandiflorus* and *Z. mays* (JG‐YM), *P. grandiflorus* and *S. italica* (JG‐GZ), *P. grandiflorus* and *G. max* (JG‐DD). Each treatment was replicated three times, resulting in 21 plots, each with an area of 18.75 m^2^ (3 m × 6.25 m). The experiment commenced in April 2023, with *P. grandiflorus* transplanted as one‐year‐old seedlings at a row spacing of 25 cm and plant spacing of 10 cm. Other crops were seeded with the following specifications: *A. bidentata* at 25 cm row spacing and 15 cm plant spacing, *A. stricta* at 30 cm row spacing and 20 cm plant spacing, *S. divaricata* at 25 cm row spacing and 20 cm plant spacing, *Z. mays* at 30 cm row spacing and 25 cm plant spacing, *G. max* at 30 cm row spacing and 20 cm plant spacing, and *S. italica* at 25 cm row spacing and 10 cm plant spacing. The normal field management practices were applied throughout the study period. The root samples of *P. grandiflorus* were collected during the seedling stage (the first sampling), vigorous growth period (the second sampling), and optimal harvesting period (the third sampling). Each plot employed a 5‐point sampling method, with 10–15 fresh samples collected for the physiological and biochemical analyses, while the remaining roots were dried and crushed for saponin content determination.

### 2.3 Biomass Measurement

The soil from the seedlings of *P. grandiflorus* was washed with tap water six times using six plants. Subsequently, the seedlings were wiped with a filter and absorbent paper to remove excess water. Both the aboveground and underground parts of each plant were placed into the same cowhide bag and then oven‐dried at 105°C to terminate the green color. Once the oven temperature (DL‐101‐3PS, Tianjin Zhonghuan Electric Furnace Experimental Instrument Company, China) decreased, the samples were dried at 65°C until they reached a constant weight. The samples were removed, and their dry weights were recorded (Wang, Zhu, and Pan 2024).

### 2.4 Chlorophyll Content Determination

The chlorophyll content in the leaves was determined using the Lynd intelligent chlorophyll analyzer (LD‐YE ± 0.3 SPAD, Shandong Leiente Intelligent Technology Co. Ltd., China). For each sampling period, three *P. grandiflorus* plants from each plot were selected, and three healthy leaves from each plant were measured (Dąbrowski et al. 2024).

### 2.5 Determination of Antioxidant Enzymes and Malondialdehyde Content

The malondialdehyde (MDA) content was determined using the thiobarbituric acid method (Heath and Packer 1968). The superoxide dismutase (SOD) activity was measured using the nitrogen blue tetrazolium (NBT) method (Minami and Yoshikawa 1979). The peroxidase (POD) activity was assessed using the guaiacol method (Castro et al. 2017). The catalase (CAT) activity was measured using the ultraviolet absorption method (Berger 2010).

### 2.6 Determination of Osmotic Regulatory Substances in *P. grandiflorus*

The proline content was determined using the acidic ninhydrin method (Bates 1976). The soluble sugar content was measured using the anthrone colorimetric method (Irigoyen et al. 2010). The soluble protein content was assessed using the Coomassie Brilliant Blue method (Kielkopf et al. 2020).

### 2.7 Determination of Contents of Five Saponin Components in *P. grandiflorus*

The HPLC‐ELSD (1260 infinity II, Agilent Technologies Co. Ltd., America) method was utilized to quantify the five saponins: platycodin D3, deapoplatycodin D, platycodin D, onjisaponin, and platyconic acid (Kwon et al. 2017). The analysis utilized a Hypersil GOLD C18 selective HPLC column (4.6 mm × 250 mm, 5 μm). The mobile phase comprised 1% formic acid aqueous solution (A) and acetonitrile (B), with gradient elution conditions as follows: 0–15 min, 80%–78% A; 15–60 min, 78%–78% A; 60–65 min, 78%–70% A; 65–70 min, 70%–80% A; and 70–80 min, 80%–0% A. The flow rate was 1.0 mL·min^−1^, and the column temperature was maintained at 35°C. The ELSD detection was performed with a drift tube temperature of 110°C and a gas flow rate of 2.5 L·min^−1^.

### 2.8 Data Analysis

The statistical analyses and data plotting were performed using Microsoft Excel 2019 and GraphPad Prism 9 (2018). The significant differences were analyzed using SPSS 22.0. The comprehensive competitiveness was assessed using the principal component analysis (PCA).

## 3 Results

### 3.1 Roots Biomass of *P. grandiflorus* at Different Growth Stages

The results indicated the significant differences in the biomass between intercropping systems and monoculture modes. The measurements of seedling height, root length, aboveground fresh weight, underground fresh weight, aboveground dry weight, and underground dry weight of *P. grandiflorus* under various intercropping modes are presented in Table 1. In the first sampling, the intercropping treatment JG‐DD significantly increased the root length, aboveground fresh weight, and underground fresh weight compared to the monoculture JG‐JG treatment, with the notable increases in the root length (from 20.58 to 24.05 cm) and the aboveground fresh weight (from 13.02 to 14.87 g/plant). No significant differences were observed between other intercropping modes and the control group, nor in the seedling height among the treatments. In the second sampling, JG‐NX exhibited the highest values for most indicators, except the root length, followed by JG‐DD and JG‐SS. In the third sampling period, the JG‐NX and JG‐DD treatments significantly increased the seedling height, and all the intercropping treatments resulted in the significant increases in the root length. The fresh weights of the aboveground parts, underground parts, and dry weights of both parts were significantly higher under JG‐DD, with the increases of 28.88%, 16.43%, 23.78%, and 13.86%, respectively. Overall, the biomass indicators for JG‐DD, JG‐NX, and JG‐SS demonstrated better performance, indicating that these intercropping modes enhanced the growth of *P. grandiflorus* .

**TABLE 1:** Effects of different intercropping patterns on the biomass of *P. grandiflorus* at different growth stages.

Growth indexes							
Sampling times	Treatment	Seedling height (cm)	Root length (cm)	Aboveground part fresh weight (g/plant)	Underground part fresh weight (g/plant)	Aboveground plant dry weight (g/plant)	Underground plant dry weight (g/plant)
The first sampling	JG‐JG	55.46 ± 7.13^ab^	20.58 ± 1.63^b^	13.02 ± 3.84^b^	9.87 ± 1.86^b^	3.07 ± 0.89^c^	2.91 ± 0.75^c^
	JG‐NX	58.74 ± 0.68^a^	21.91 ± 3.09^ab^	14.45 ± 1.66^a^	11.88 ± 1.91^a^	3.76 ± 0.63^a^	2.86 ± 0.36^c^
	JG‐FF	51.61 ± 3.08^b^	20.48 ± 1.40^b^	12.41 ± 0.75^c^	10.81 ± 2.02^ab^	3.03 ± 0.09^c^	3.02 ± 0.18^b^
	JG‐SS	55.01 ± 6.19^ab^	19.67 ± 0.44^c^	13.61 ± 3.62^b^	9.79 ± 2.06^b^	3.35 ± 1.22^b^	2.83 ± 0.63^c^
	JG‐YM	52.71 ± 2.40^b^	20.26 ± 3.58^b^	14.33 ± 2.34^b^	9.63 ± 1.25^b^	3.59 ± 0.59^ab^	3.47 ± 0.41^b^
	JG‐GZ	52.66 ± 3.57^b^	21.45 ± 2.14^ab^	12.83 ± 1.67^c^	10.49 ± 0.30^ab^	3.06 ± 0.55^c^	3.07 ± 0.06^b^
	JG‐DD	51.68 ± 1.36^b^	24.05 ± 0.42^a^	14.87 ± 1.31^a^	11.79 ± 1.94^a^	3.72 ± 0.38^a^	3.51 ± 0.64^a^
The second sampling	JG‐JG	69.27 ± 2.92^ab^	22.81 ± 1.70^b^	19.95 ± 5.83^b^	15.45 ± 2.42^c^	6.12 ± 1.60^c^	4.35 ± 0.76^b^
	JG‐NX	75.87 ± 3.60^a^	22.60 ± 0.38^b^	27.57 ± 0.65^a^	20.61 ± 0.52^a^	8.72 ± 1.31^a^	6.29 ± 0.39^a^
	JG‐FF	63.66 ± 6.79^c^	20.87 ± 1.68^c^	21.00 ± 14.27^b^	17.08 ± 9.15^b^	6.05 ± 3.74^c^	4.80 ± 2.11^b^
	JG‐SS	68.19 ± 17.30^b^	23.08 ± 1.47^ab^	24.01 ± 9.96^ab^	17.67 ± 2.28^b^	7.54 ± 3.89^b^	5.26 ± 0.82^ab^
	JG‐YM	69.89 ± 8.14^ab^	21.35 ± 1.54^b^	17.43 ± 4.31^c^	13.25 ± 3.64^d^	5.46 ± 2.08^d^	4.21 ± 0.80^b^
	JG‐GZ	63.34 ± 10.61^c^	20.46 ± 2.46^c^	17.22 ± 3.80^c^	14.49 ± 2.77^c^	5.51 ± 1.51^d^	4.32 ± 0.74^b^
	JG‐DD	69.94 ± 5.06^a^	23.93 ± 2.58^a^	25.33 ± 2.17^a^	20.84 ± 1.32^a^	8.02 ± 0.88^ab^	6.22 ± 0.18^a^
The third sampling	JG‐JG	64.49 ± 3.20^b^	21.38 ± 0.15^c^	13.61 ± 1.03^b^	21.10 ± 1.40^b^	5.48 ± 0.59^b^	6.65 ± 0.70^b^
	JG‐NX	71.76 ± 6.66^a^	26.08 ± 1.91^a^	21.79 ± 6.14^a^	20.78 ± 4.26^b^	7.98 ± 1.42^a^	8.44 ± 1.21^a^
	JG‐FF	63.42 ± 3.47^b^	22.72 ± 0.82^b^	12.68 ± 7.10^b^	19.03 ± 6.93^b^	5.02 ± 2.49^b^	6.48 ± 1.38^b^
	JG‐SS	64.25 ± 13.75^b^	24.88 ± 5.20^ab^	15.73 ± 7.44^b^	23.96 ± 3.77^a^	6.07 ± 2.21^ab^	6.76 ± 2.16^b^
	JG‐YM	64.32 ± 12.49^b^	23.08 ± 2.71^b^	9.24 ± 3.99^c^	17.65 ± 5.70^c^	4.06 ± 1.99^b^	5.86 ± 1.90^c^
	JG‐GZ	63.83 ± 9.08^b^	22.50 ± 2.66^b^	8.45 ± 4.84^c^	14.67 ± 2.38^d^	3.97 ± 1.92^c^	4.93 ± 1.00^c^
	JG‐DD	69.40 ± 0.39^a^	24.46 ± 1.25^ab^	19.12 ± 6.03^a^	25.25 ± 5.08^a^	7.19 ± 2.69^a^	7.72 ± 1.38^a^

*Note:* The significance difference analysis in the table is based on the comparison of data in the same column, with JG‐JG continuous cropping mode as the control, and the lowercase letters (a, b, c) in the same column indicate significant differences at the 5% level between the same period treatments (*p* < 0.05).

### 3.2 Chlorophyll Content of *P. grandiflorus* Leaves at Different Growth Stages

**FIGURE 1:**
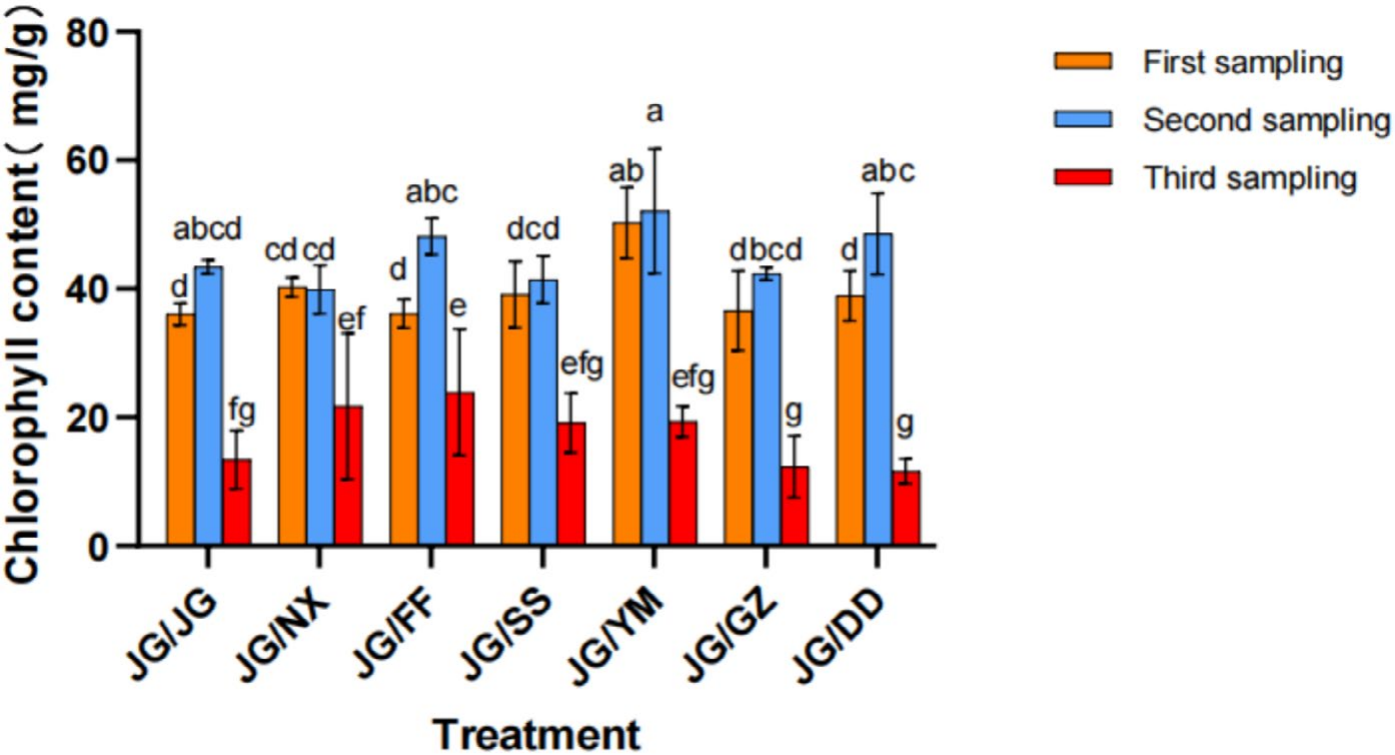
The effects of different intercropping patterns on chlorophyll content of *P. grandiflorus* at different growth stages activity. Different letters indicate significant differences between treatments (*p* < 0.05), the same below.

In the first sampling, the chlorophyll content for the JG‐JG treatment was the lowest among the seven treatments, while the JG‐YM treatment exhibited the highest chlorophyll content at 50.26 mg·g^−1^. In the second sampling, JG‐YM maintained the highest chlorophyll content, followed by JG‐DD, which showed the increases of 16.64% and 10.56%, respectively, compared with the JG‐JG control group. However, neither treatment significantly differed from the control group (*p* > 0.05). In the third sampling, the chlorophyll content significantly decreased across all the treatments. Overall, the chlorophyll indicators in JG‐YM, JG‐DD, and JG‐FF were superior (Figure 1).

### 3.3 Content of Malondialdehyde (MDA) in Roots of *P. grandiflorus* at Different Growth Stages

**FIGURE 2:**
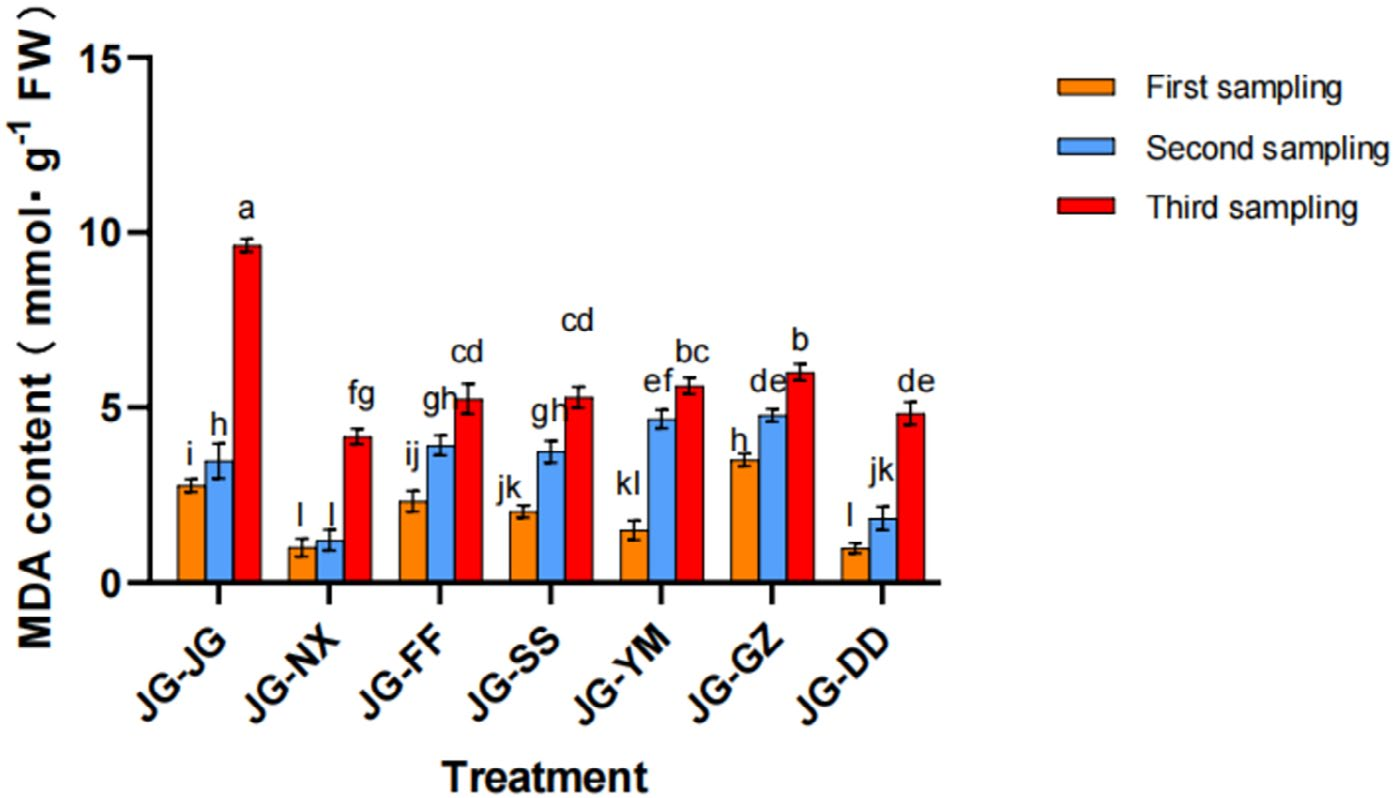
The effects of different intercropping modes on the content of malondialdehyde (MDA) in *P. grandiflorus* at different growth stages.

As sampling time increased, the MDA content increased progressively under each treatment. In the third sampling, the JG‐JG treatment exhibited the highest MDA content, whereas other six treatments demonstrated the significantly reduced levels compared to JG‐JG. Among these, JG‐NX had the lowest MDA content, with the reductions of 63.75%, 65.21%, and 56.70% compared to JG‐JG, followed by JG‐DD. The results indicated that the MDA content remained relatively stable under the JG‐NX and JG‐DD treatments throughout the three sampling periods, demonstrating strong stability (Figure 2).

### 3.4 Antioxidant Enzymes in Roots of *P. grandiflorus* at Different Growth Stages

**FIGURE 3:**
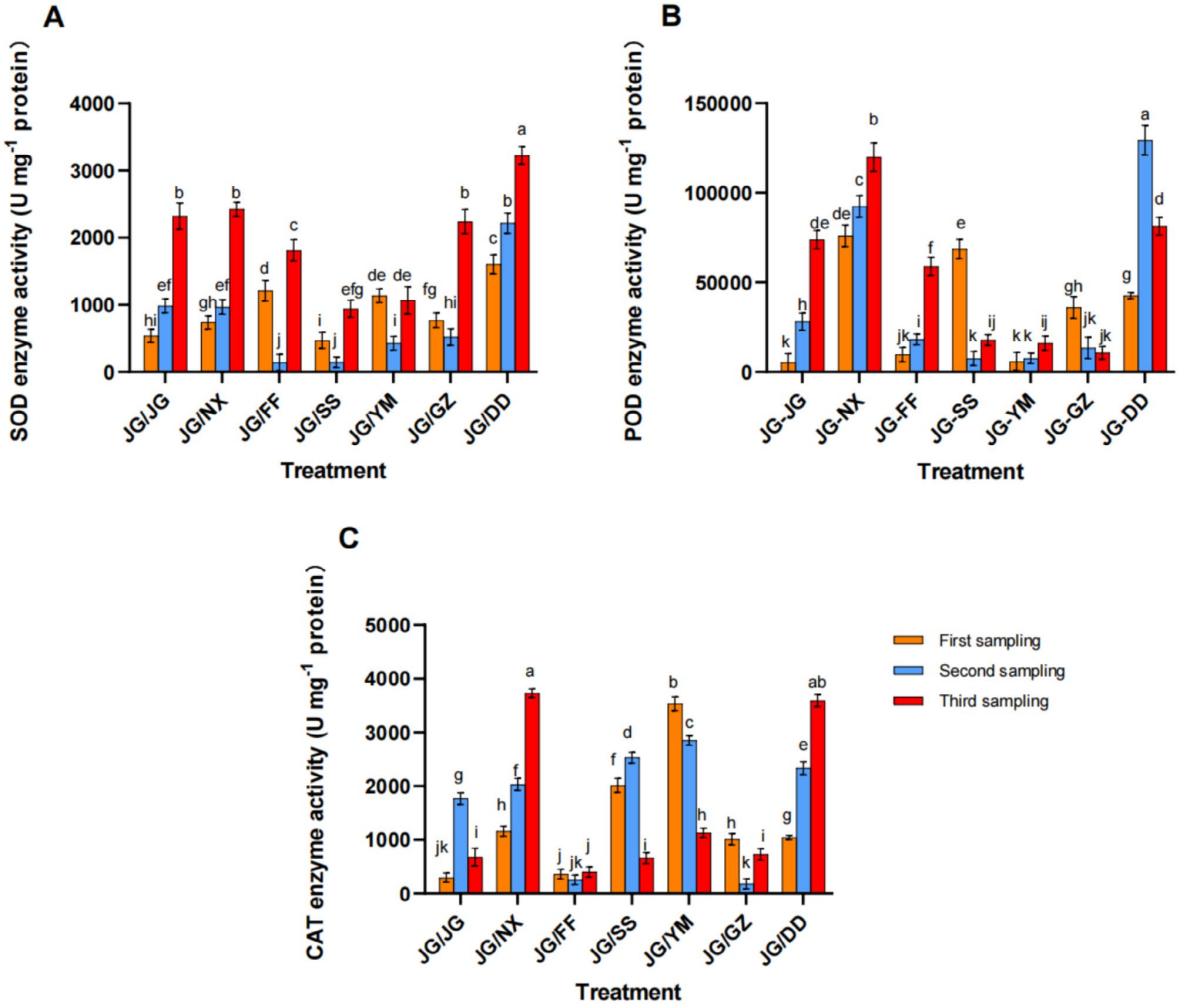
The effects of different intercropping modes on antioxidant enzymes in *P. grandiflorus* at different growth stages. (A) The superoxide dismutase (SOD) activity, (B) the peroxidase (POD) activity, (C) the catalase (CAT) activity.

Overall, the SOD enzyme activity exhibited an upward trend in the JG‐JG, JG‐NX, and JG‐DD treatments, while the JG‐FF, JG‐SS, JG‐YM, and JG‐GZ treatments initially decreased and then increased. The JG‐DD treatment demonstrated a significant overall increase compared to JG‐JG, with the SOD enzyme activity being the highest among the seven treatments in the first and second sampling, presenting the increases of 83.38% and 55.51%, respectively, compared to JG‐JG (Figure 3A). A gradual increase in the POD content helped mitigate the damage from elevated peroxides and decelerated the plant aging. In the first sampling, the POD enzyme activity was the highest under JG‐NX, followed by JG‐SS. In the second sampling, JG‐DD exhibited the highest POD enzyme activity, which increased by 78.34% compared to JG‐JG. In the third sampling, the highest POD content was observed in JG‐NX (Figure 3B). CAT contributed to the antioxidant enzyme system in plants and offered the protective benefits. Compared to JG‐JG, the JG‐NX, JG‐YM, and JG‐DD treatments had significantly higher CAT enzyme activities. In the third sampling period, the highest CAT activity was observed in JG‐NX, followed by JG‐DD (Figure 3C). Overall, the antioxidant enzyme activity of *P. grandiflorus* roots was significantly higher in the JG‐NX, JG‐DD, JG‐SS, and JG‐YM treatments than in the JG‐JG control, without significant difference between the JG‐GZ and JG‐FF treatments.

### 3.5 Osmotic Regulation Content in Roots of *P. grandiflorus* at Different Growth Stages

**FIGURE 4:**
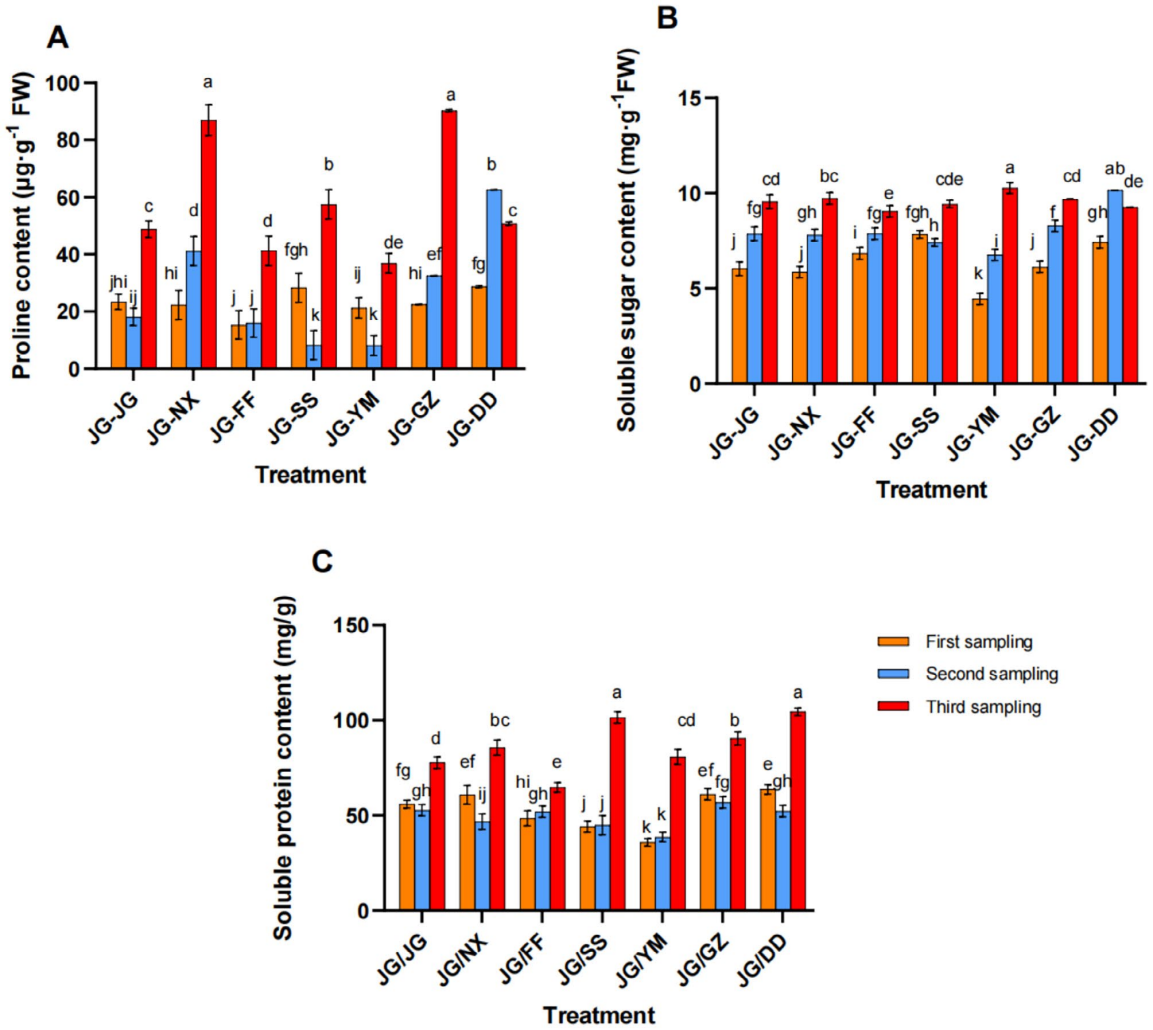
The effects of different intercropping modes on the osmotic regulatory substance content in roots of *P. grandiflorus* at different growth stages. (A) The proline content, (B) the soluble sugar content, (C) the soluble protein content.

With the extension of sampling time, the proline content significantly increased under each treatment. In the third sampling, the highest proline content was observed in the JG‐NX and JG‐GZ treatments, followed by JG‐SS, with the increases of 43.94%, 46.01%, and 15.32%, respectively, compared with the JG‐JG treatment (Figure 4A). The overall change in soluble sugar content was not significant compared to the JG‐JG treatment. In the second sampling, the JG‐DD treatment exhibited the highest soluble sugar content, with the 22.53% increase compared to JG‐JG. In the third sampling, the highest soluble sugar content was identified in the JG‐YM treatment, which increased by 7.01% compared to the control group (Figure 4B). Among the different intercropping modes, the soluble protein content generally increased over time, with the JG‐SS, JG‐DD, and JG‐NX treatments demonstrating more significant increases. In the third sampling, these treatments exhibited the increases of 23.38%, 25.59%, and 9.20%, respectively, compared to JG‐JG (Figure 4C).

### 3.6 Five Saponin Components in Roots of *P. grandiflorus* at Different Growth Stages

**FIGURE 5:**
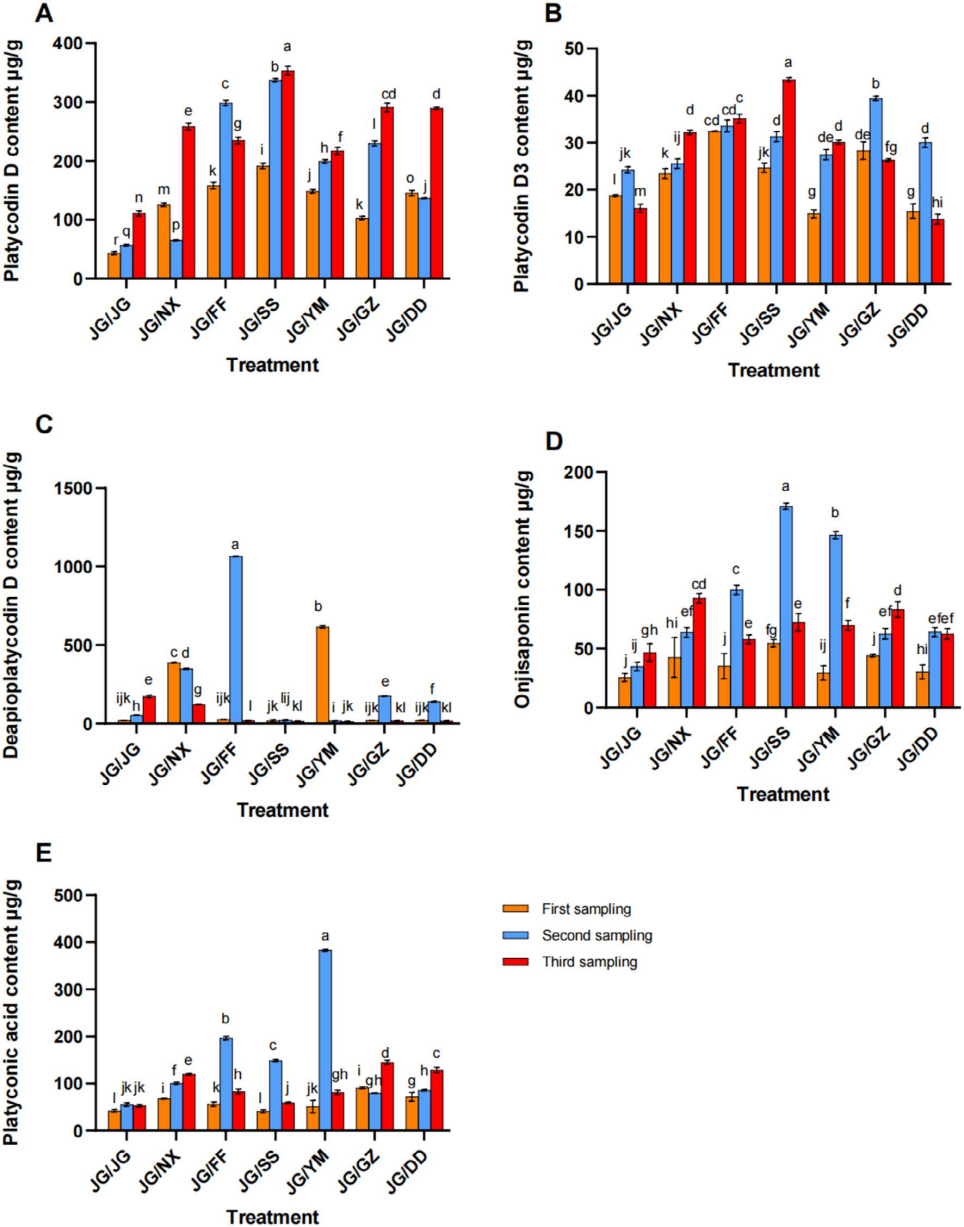
The effects of different intercropping methods on the five saponin components of *P. grandiflorus* at different growth stages. (A) The platycodin D content, (B) the platycodin D3 content, (C) the deapoplatycodin D content, (D) The onjisaponin content, (E) the platyconic acid content.

The results indicated that different intercropping methods could enhance the content of various platycodon saponins, with platycodin D being the primary active ingredient of *P. grandiflorus* . Except for the JG‐FF treatment group, all other treatments significantly increased platycodin D content during the harvest period. Specifically, compared to the JG‐JG treatment, the JG‐NX, JG‐SS, JG‐YM, JG‐GZ, and JG‐DD treatments increased the platycodin D content by 57.09%, 68.91%, 48.90%, 61.93%, and 61.69%, respectively (Figure 5A). Furthermore, the platycodin D3 content significantly increased in the JG‐NX, JG‐FF, JG‐SS, and JG‐GZ treatments compared to that in JG‐JG. In the third sampling, JG‐SS presented the highest platycodin D3 content among the seven treatments, with the 62.96% increase compared to the control group, followed by JG‐FF, which increased by 54.24% compared to the control group (Figure 5B).

The deapoplatycodin D content was measured under different intercropping modes. In the first sampling, JG‐YM resulted in the highest deapoplatycodin D content in the *P. grandiflorus* roots, with the 96.67% increase compared to the control group, followed by JG‐NX, with a 94.71% increase. The contents in JG‐SS and JG‐DD were lower than that in the control group. In the second sampling, JG‐FF had the highest deapoplatycodin D content among the treatments, with the 95.03% increase compared to the control group. In the third sampling, the JG‐JG control group showed the highest deapoplatycodin D content among all treatments (Figure 5C).

Overall, the onjisaponin content was higher in all the six intercropping treatments than in the control group. In the second sampling, JG‐SS had the highest onjisaponin content among the seven treatments, with the 79.60% increase compared to the control group, followed by the JG‐YM treatment (Figure 5D).

The results suggested that compared to the JG‐JG treatment, the content of platyconic acid in the roots of *P. grandiflorus* increased under all the six intercropping modes. In the second sampling, JG‐YM exhibited the highest platyconic acid content among the seven treatments, with an 85.70% increase compared to the control group, followed by the JG‐FF and JG‐SS treatments (Figure 5E).

### 3.7 Comparison of Comprehensive Competitiveness of Multi‐Index Components

**FIGURE 6:**
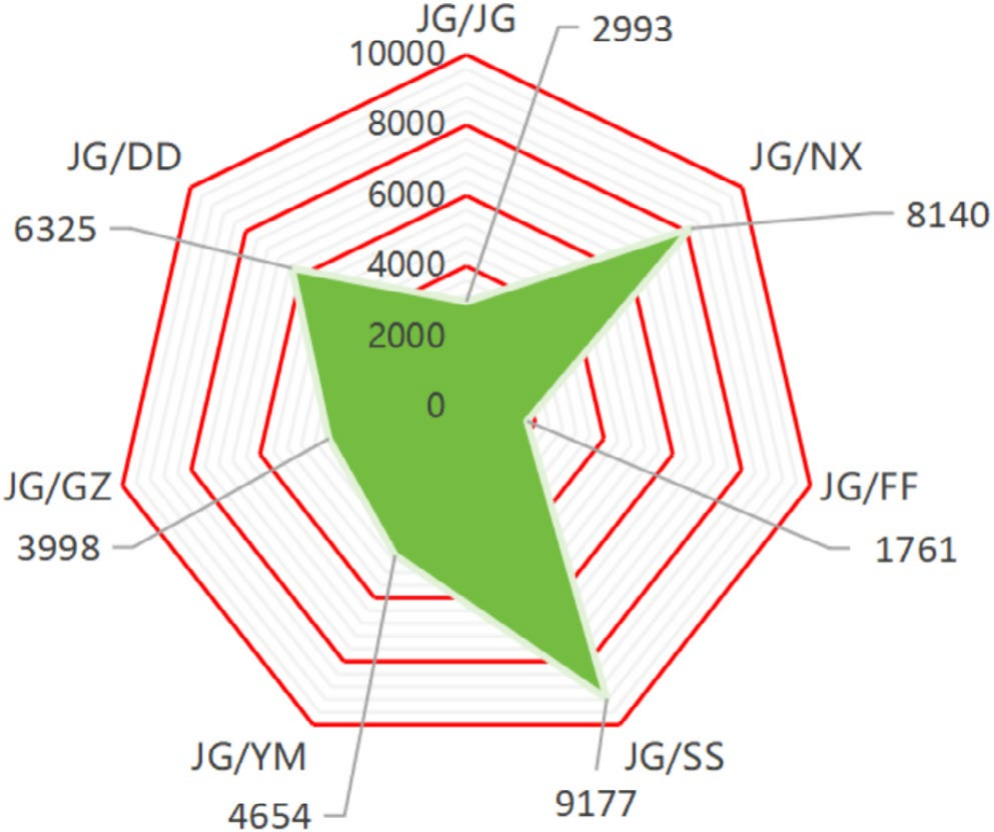
The comprehensive competitiveness of multi index components. 0–10,000 represents the score of comprehensive competitiveness under different treatment groups. The green part represents the proportion of comprehensive competitiveness, and the green area is positively correlated with comprehensive competitiveness.

The principal component analysis (PCA) was performed to derive a comprehensive score based on two indicators: the component score and variance explained rate, which incorporated the biomass, physiological, and biochemical indicators, and five saponin components to evaluate the overall competitiveness. The results revealed that the comprehensive competitiveness score was the highest for the JG‐SS treatment, followed by JG‐NX and JG‐DD, with the scores of 9177, 8140, and 6325, respectively. The JG‐FF treatment exhibited the lowest comprehensive competitiveness score (Figure 6).

## 4 Discussion

Intercropping can enhance the ecosystem balance and stability, promote the interactions between different plants, improve the plant quality, and increase the resistance to diseases and stress (Zhu et al. 2024). This study utilized various intercropping modes to measure the biomass, chlorophyll, antioxidant enzyme levels, osmoregulatory substance content, and saponin components of *P. grandiflorus* at different growth and development stages, offering diverse insights into the effects of intercropping on plants.

Plant biomass represents the total amount of organic matter per unit area at a specific time and is a crucial parameter for studying vegetation production, carbon cycling, and nutrient allocation in terrestrial ecosystems (Roberts et al. 1985). Research has indicated that intercropping rice with banana can reduce the rice plant height during the late tillering and heading stages of early rice, while increasing the chlorophyll content in rice leaves during the early, mature, and late tillering stages (Shahidullah et al. 2024). In our study, intercropping *P. grandiflorus* with other crops led to the increases in the root length, aboveground fresh weight, belowground fresh weight, and aboveground dry weight. However, the differences were observed at various growth stages. During the seedling stage, only the JG‐DD treatment group demonstrated the significant improvements in various indicators compared with the control group, whereas other intercropping treatments did not exhibit significant changes. This may be attributed to the short duration of intercropping, as the effects between crops were not fully understood. With the prolonged intercropping time, the effects became more pronounced, as reflected in the superior biomass indicators of JG‐DD, JG‐NX, and JG‐SS compared to other treatments.

The chlorophyll content is a crucial indicator for diagnosing crop nutrition and studying photosynthesis. Higher chlorophyll content in plant leaves can enhance the photosynthetic capacity and promote the greater dry matter accumulation. The three‐dimensional structure created by intercropping can significantly improve temperature, humidity, and lighting conditions, which directly affects chlorophyll synthesis (Xhu et al. 2018). Intercropping *Avena sativa* with *Vicia sativa* can increase the chlorophyll content of both species (Wang, Zhu, and Pan 2024). Similarly, our study discovered that intercropping *P. grandiflorus* with various crops generally increased the chlorophyll content in its leaves, with the JG‐YM treatment demonstrating the highest chlorophyll levels. This improvement could be due to the reduced high temperatures and intense light in intercropping systems compared to the monoculture areas, which mitigated the stress‐induced decline in chlorophyll synthesis (Szymańska et al. 2017).

MDA can damage the cell membrane system, proteins, and DNA, leading to the membrane degradation and the loss of cell function. Consequently, the MDA content can reflect the extent of oxidative damage to plants (Bao et al. 2020). In this study, as the cultivation cycle progressed, the MDA content in various *P. grandiflorus* intercropping treatments generally decreased, indicating a strong stability.

Plant antioxidant enzymes are crucial biomolecules that help eliminate reactive oxygen species and mitigate oxidative stress in plants. SOD widely distributed in plants can catalyze the dismutation of superoxide anion radicals into oxygen and hydrogen peroxide and thus plays a vital role in maintaining the balance between the oxidation and antioxidant activities (Zhao et al. 2024). CAT, along with SOD and POD, forms the plant antioxidant enzyme system and provides protection (Huang et al. 2023). POD is involved in various physiological processes and produces the hydrolytic enzymes that resist pathogens, inhibit the reproduction, and enhance the plant disease resistance (Cai et al. 2021). Previous research has demonstrated that the POD, SOD, and CAT enzyme activities in *Oryza sativa* leaves increase under intercropping compared to monoculture. In this study, the overall antioxidant enzyme activity was higher under the JG‐NX and JG‐DD treatments.

Osmotic regulation is crucial for plant growth and development (Xu et al. 2020). Investigations have indicated that continuous cropping obstacles can lead to the deficiencies in multiple trace elements in *Melilotus officinalis* , which can restrict its growth and reproduction (Zeeshan Ul Haq et al. 2023). The reduction in soil elements can cause lower osmotic potential, creating osmotic stress in plants (Zhao et al. 2021). Plants mitigate this stress by synthesizing osmotic regulatory substances, such as proline, soluble sugars, and soluble proteins (Ozturk et al. 2021). In addition, studies have demonstrated that intercropping *Prunus triloba* with *Beta vulgaris* can enhance the accumulation of soluble sugars and proteins in *Beta vulgaris* leaves, with the increases in the soluble proteins, proline, and soluble sugars observed as intercropping time increases (Li et al. 2021). Consistent with these findings, our study indicated that various intercropping modes generally increased the content of osmoregulatory substances in *P. grandiflorus* at different growth stages, where certain differences among treatments were observed. Overall, the intercropping modes JG‐NX, JG‐SS, and JG‐DD effectively mitigated the damage caused by continuous cropping and promoted the growth and development of *P. grandiflorus* plants.

Saponins are a major class of active ingredients in the roots of *P. grandiflorus* , and triterpenoid saponins are the primary compounds studied in plants. Their biosynthesis has been extensively studied (Nyakudya et al. 2014). *P. grandiflorus* , certain triterpenoid saponins such as platycodin D3, deapioplatycodin D, and platycodin D are present, with platycodin D being the principal active ingredient. Platycodin D can exhibit various pharmacological effects, including anti‐inflammatory, hypoglycemic, lipid‐lowering, antitumor, immune‐regulating, and anti‐allergic properties (Jolly et al. 2024). Onjisaponin and platyconic acid also possess pharmacological effects, such as antioxidant and anti‐aging properties (Zhang et al. 2015). In this study, the content of platycodin D3, platycodin D, onjisaponin, and platyconic acid in the roots of *P. grandiflorus* significantly increased after the JG‐SS and JG‐YM treatments, suggesting that these treatments enhanced the quality of medicinal materials. This finding aligned with previous studies. However, in the JG‐SS, JG‐DD, and JG‐GZ treatments, the deapoplatycodin D content was notably reduced. This reduction may be attributed to the alterations in the downstream biosynthetic pathways of platycodin D, D3, and deapoplatycodin D, most of which can be synthetic (Ma et al. 2016). Further research is needed to elucidate the mechanisms behind the low content of deapoplatycodin D.

## 5 Conclusions

This study thoroughly examined the effects of various intercropping modes on *P. grandiflorus* across different growth and development stages, offering valuable insights into the standardized cultivation of *P. grandiflorus* with other crops. The results demonstrated that intercropping enhanced the biomass indicators, increased the chlorophyll content, reduced the MDA content, and elevated both the antioxidant enzyme activity and osmoregulatory substance content at various growth stages of *P. grandiflorus* . Moreover, the overall content of platycodon saponin components, particularly platycodin D, was higher in intercropping modes, indicating that intercropping with different crops effectively improved the *P. grandiflorus* quality. It was recommended to intercrop *P. grandiflorus* with *A. stricta* (JG‐SS), *A. bidentata* (JG‐NX), *G. max* (JG‐DD), *Z. mays* (JG‐YM), and *S. italica* (JG‐GZ) in production practices, with *A. stricta* providing the best results for achieving maximum yield and optimal quality. Conversely, intercropping *P. grandiflorus* with *S. divaricata* was not recommended.

## Conflicts of Interest

The authors declare no conflicts of interest.

## Data Availability

All data needed to evaluate the conclusions in the paper are present in the paper and/or the Supporting Information.
